# Supplementary material for: Causal Relationship between Aging and Anorexia Nervosa: A White-Matter-Microstructure-Mediated Mendelian Randomization Analysis
Source: Biomedicines. 2024 Aug 16;12(8):1874. doi: 10.3390/biomedicines12081874 (PMC11351342; doi:10.3390/biomedicines12081874)
Supplement: Supplementary file 1 [file biomedicines-12-01874-s001.zip › Supplementary Figure/Figure S8/Leaveoneout plot of MR analysis for TL to ubm-b-1565.pdf]

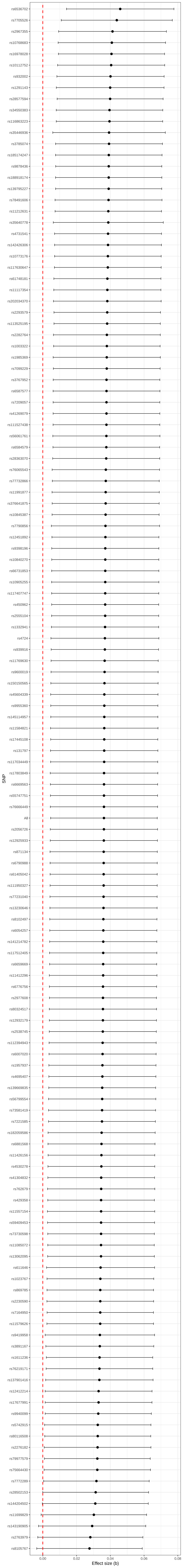

rs6536702
rs7705526
rs2967355
rs10768683
rs16978028
rs10112752
rs932002
rs1291143
rs28577594
rs34550383
rs116863223
rs35446936
rs3785074
rs185174247
rs9878436
rs188918174
rs139795227
rs78491606
rs11212631
rs35640778
rs4731541
rs142426306
rs10773176
rs117630647
rs61748181
rs11117354
rs202034370
rs2293579
rs113525195
rs2282764
rs1003322
rs1985369
rs7099229
rs3767952
rs6587577
rs7209057
rs41269079
rs111527438
rs56061761
rs6584579
rs28363070
rs76065543
rs77732866
rs11991877
rs376641875
rs10845387
rs7790856
rs12451892
rs9398196
rs10840270
rs66731853
rs10905255
rs117407747
rs450962
rs2555104
rs1332941
rs4724
rs939916
rs11769630
rs9600019
rs150150565
rs45604339
rs9955360
rs145114957
rs11584821
rs17445108
rs131797
rs117034449
rs17803849
rs6669563
rs55747751
rs76666449
All
rs2056726
rs12925933
rs871134
rs6790988
rs61405042
rs111950327
rs77231040
rs13230646
rs8102497
rs6054257
rs141214782
rs117512405
rs6659669
rs11412296
rs6776756
rs2977608
rs80324517
rs12932179
rs2538745
rs112394943
rs6007020
rs1957937
rs4695407
rs139669835
rs56799554
rs73581419
rs7221585
rs182059586
rs6881568
rs11426156
rs4530278
rs41304832
rs762679
rs429358
rs11557154
rs59409453
rs73730598
rs11085072
rs13062095
rs611646
rs1023767
rs869785
rs2230590
rs7164950
rs11579626
rs9419958
rs3891167
rs1611236
rs76219171
rs137901416
rs12412214
rs17677991
rs9940099
rs5742915
rs80116508
rs2276182
rs79977579
rs75664430
rs7772289
rs28502153
rs144204502
rs11699829
rs143190905
rs2763979
rs8105767
SNP
0.00
0.02
0.04
0.06
0.08
Effect size (b)
